# An Ultrahigh Sensitive Paper-Based Pressure Sensor with Intelligent Thermotherapy for Skin-Integrated Electronics

**DOI:** 10.3390/nano10122536

**Published:** 2020-12-17

**Authors:** Lin Gao, Junsheng Yu, Ying Li, Peiwen Wang, Jun Shu, Xiaoyan Deng, Lu Li

**Affiliations:** 1 State Key Laboratory of Electronic Thin Films and Integrated Devices, School of Optoelectronic Science and Engineering, University of Electronic Science and Technology of China (UESTC), Jianshe North Road, Chengdu 610054, China; lin_gaoedu@163.com (L.G.); jsyu@uestc.edu.cn (J.Y.); 2 Research Institute for New Materials Technology, Chongqing University of Arts and Sciences, Honghe Avenue, Chongqing 402160, China; pwenwang@163.com (P.W.); s917549966@163.com (J.S.); dxy151234@163.com (X.D.)

**Keywords:** pressure sensors, tissue, thermotherapy, wearable electronics

## Abstract

Porous microstructure pressure sensors that are highly sensitive, reliable, low-cost, and environment-friendly have aroused wide attention in intelligent biomedical diagnostics, human–machine interactions, and soft robots. Here, an all-tissue-based piezoresistive pressure sensor with ultrahigh sensitivity and reliability based on the bottom interdigitated tissue electrode and the top bridge of a microporous tissue/carbon nanotube composite was proposed. Such pressure sensors exhibited ultrahigh sensitivity (≈1911.4 kPa^−1^), fast response time (<5 ms), low fatigue of over 2000 loading/unloading cycles, and robust environmental degradability. These enabled sensors can not only monitor the critical physiological signals of the human body but also realize electrothermal conversion at a specific voltage, which enhances the possibility of creating wearable thermotherapy electronics for protecting against rheumatoid arthritis and cervical spondylosis. Furthermore, the sensor successfully transmitted wireless signals to smartphones via Bluetooth, indicating its potential as reliable skin-integrated electronics. This work provides a highly feasible strategy for promoting high-performance wearable thermotherapy electronics for the next-generation artificial skin.

## 1. Introduction

Pressure sensors, which convert pressure signals into electrical signals, offer a variety of existing and emerging applications, such as soft robots, human–machine interfaces, medical diagnostics, and artificial intelligences, among many others [1,2,3,4,5,6]. For instance, such sensors can be spontaneously mounted on a dynamic and curved epidermis to detect the body’s vital physiological mechanical signals through real-time transmissions, thus realizing wearable Internet of Things in society [7,8,9,10]. At present, piezoelectric [11], piezoresistive [12,13], triboelectric [14,15], and capacitive [16,17] pressure sensing mechanisms and functional materials with multivariant microstructure collocation strategies have converged to meet the requirements of next-generation wearable electronics. Taking the manufacturing cost, sensitivity, response time, reliability, and large-area array process into consideration, a piezoresistive device is regarded as an attractive choice for next-generation pressure sensors.

In recent years, substantial breakthroughs have been made in relevant research, the first is to select various highly conductive materials as pressure-sensing elements, such as conductive polymers [18,19,20], carbon materials [21,22,23,24,25,26], metal nanowires [27,28,29], and two-dimensional materials [30]. The second is to design sophisticated microstructures to induce conductive contact enhancement, mainly including common porous structures [31], micropyramid structures [32], microdome structures [33], micropillar structures [34], and so forth. Although these microstructures have been optimized and improved continuously by multiple technologies, the complicated harsh and time-consuming fabrication processes are always neglected, limiting their practical applications to a certain extent. Consequently, pressure sensors with a facile, low-cost, and environment-friendly method must be dominant choices, and one feasible approach to realize that is through paper-based wearable electronic devices [35,36]. The natural properties and porous microstructure of the paper provide the basic conditions for the realization of high-performance pressure sensors [37]. Some recent studies demonstrated that the conductive materials of a paper-based pressure sensor were embedded into the microstructure fiber paper using various preparation processes [38,39,40,41,42,43,44,45,46]. For example, Tao et al. proposed a pressure sensor fabricated by mixing the tissue paper with graphene oxide solution, whose performance indicated a pressure range of 20 kPa and a high sensitivity of 17.2 kPa^−1^ (0−2 kPa) [41]. Guo et al. prepared a sensitive and degradable pressure sensor by sandwiching a porous Ti_3_C_2_T_x_ tissue paper between a biodegradable polylactic acid (PLA) and an interdigitated electrode-coated PLA [42]. Similarly, Han et al. stacked the irregular surface and fiber network structure of multilayer carbon black and air-laid paper to increase the number of electrical contact points under pressure, which contributed to prominent performances of the pressure sensors [43]. Even if great progress was made in paper-based pressure sensors, the application is more limited to the detection of basic physiological signals and lacks depth extension, incapable of combining electrothermal properties with mechanical durability for thermotherapy application. Therefore, simultaneously achieving reliable pressure integrity and competent electrothermal properties for fabricating multifunctional smart paper-based electronics for both physiological signal monitoring and thermotherapy application is highly desired, but it remains challenging.

In this paper, we introduced a low-cost, environment-friendly, skin-comfortable, and air-permeable soft tissue as the backbone (sensitive layer and flexible substrate) to monitor microvibration physiological signals and realize pressure-regulated intelligent thermotherapy applications. The ultrahigh sensitivity and fast response time realized by the immersion process of tissue and aqueous carbon nanotube (CNT) solutions are the best results reported for paper-based pressure sensors. The spontaneous interaction between CNTs and paper fibers contributes to the formation of reliable conductive networks and robust mechanical properties. Finally, an optimized sensor was deployed on a Bluetooth module that transmits wireless signals to a smartphone. This work opens up more possibilities for practical applications in soft robots, electronic skins, intelligent healthcare, and artificial intelligence.

## 2. Experimental Section

Material Preparation: Aqueous dispersion of CNTs was purchased from XFNANO (Nanjing, China) for conductive materials. Clean and soft tissue (Figure S1a) was used as flexible substrates and the backbone of sensitive layers. Conductive sliver paste was used as bottom electrodes. Additionally, VHB film (3M VHB Tape 4910, St. Paul, MN, USA) was used to assemble the sensor. 

Device Fabrication: The soft, clean tissue was first immersed in the aqueous dispersion of CNTs for 30 s, which ensured that CNTs effectively diffused through the microporous structure and uniformly adhered to the fibers in the diffusion path. Then, the tissue/CNT composites were transferred into an oven and dried at 70 °C for 2 h to evaporate the water solvent. Meanwhile, the conductive track circuit was prepared on the flexible tissue substrate by screen printing method. To form stable and low square resistance electrodes, the silver paste was scraped three to five times along the same direction on the designed screen, followed by a drying process in the oven for 2 h. The silver electrodes coated on the tissue substrate with a sheet resistance of ≈0.42 Ω sq^−1^ are described in Figure S1. Finally, ultrathin VHB tape was used to assemble the two functional layers to form integral pressure sensors.

Measurement and Characterization: The surface morphologies of the tissue and tissue/CNT composites were characterized by field-emission SEM (GeminiSEM 300, Hallbergmoos, Germany)). The morphology of the silver paste electrode on the tissue was observed by microscopy. The 3D surface profile of the CNT-immersed tissue was characterized by laser scanning confocal microscopy (OPTELICS C130, Kanagawa, Japan). An FTIR (VERTEX 80v, Bruker, Karlsruhe, Germany) spectrometer was used to measure the infrared spectra of samples. The mechanical and electrical behaviors were analyzed via the combination of a mechanical test analyzer (TestStar ETM102B-TS, Shenzhen, China) and electrochemical workstation (CHI 760E, Shanghai, China). Additionally, a digital source meter (Keithley 2400, Beaverton, OR, USA) was used to measure the sheet resistance of the different samples.

## 3. Result and Discussion

### 3.1. Structural Design and Composition Characterization of All-Tissue Pressure Sensors

**Figure 1:**
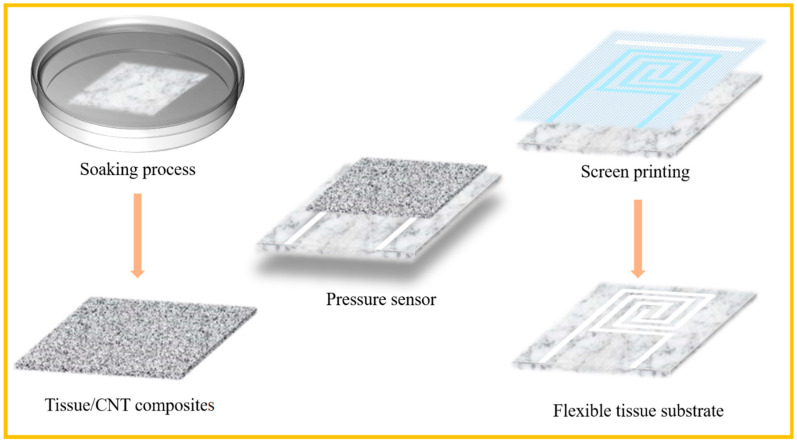
Schematic illustration of the fabrication procedure of all-tissue-based pressure sensors.

The all-soft-tissue-based pressure sensor consists of two functional layers, the top tissue/CNT composites for pressure sensing and the bottom tissue with conductive electrode acting as flexible substrate and skin contact. The fabrication procedures for the pressure sensors are illustrated in Figure 1. Such sensor not only has reliable device structure, low-cost material selection, and facile fabrication process but also can be conveniently attached to the human epidermis with skin comfortability and air breathability.

**Figure 2:**
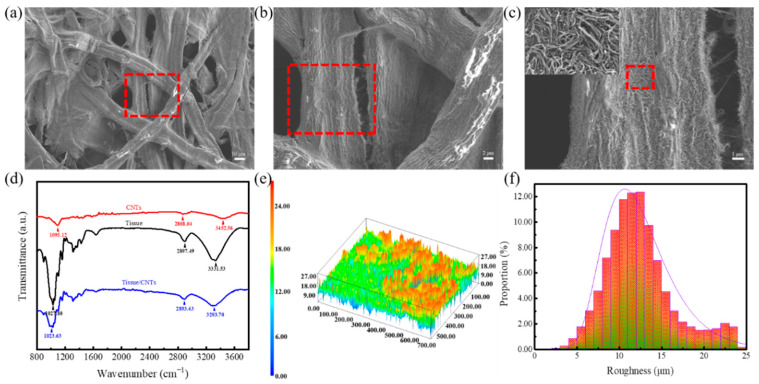
Composition characterization of the all-tissue-based pressure sensor. (**a**,**b**) Surface morphology of tissue/CNT composites. (**c**) Adhesion of CNTs to a single fiber. (**d**) FTIR results of CNT, tissue, and tissue/CNT composites. (**e**) 3D surface profile of tissue/CNT composites. (**f**) The proportion distribution of the surface roughness.

The SEM images in Figure S2 and Figure 2a–c show the surface morphologies of tissue and tissue/CNT composites. The tissue with a natural porous structure was mainly made of randomly interconnected cellulose, which facilitated the diffusion of aqueous solutions and the attachment of conductive materials (Figure S2). The uniformity of CNTs can be guaranteed through the staggered adhesion on the fibers; thus the internal porous structures and the top-down cascaded fiber contact boost the sensitivity under pressure. It is worth noting that CNTs did not cover the original porosity of the tissue during the immersion process, and the excellent air permeability contributed to the integration with human skins (Figure 2). FTIR spectroscopy was performed to reveal the chemical bonds between the tissue and CNTs (Figure 2d). The vibration peaks of the CNTs were induced by surface modification, which conduced to the stable dispersion in aqueous solution. The typical characteristic peaks of a tissue at 1027.80, 2897.49, and 3331.53 cm^−1^ can be assigned to the stretching vibration of C–O–C, C–H, and –OH groups. Additionally, a series of vibrations were red-shifted to 1023.63, 2893.43, and 3293.70 cm^−1^, indicating that a chemical interaction existed between the tissue and CNTs. Therefore, the coupling process is conducive to improving the reliability of pressure sensors. Besides, the 3D surface profile (Figure 2e,f) was further employed to reveal the overall distributions of the spiny microstructure formed by interlaced fibers, and provides a certain height range of the random distribution of the spiny microstructure for electrical contact reinforcement. 

### 3.2. Pressure-Sensing and Electrothermal-Converting Properties of the Porous Tissue-Based Sensor

**Figure 3:**
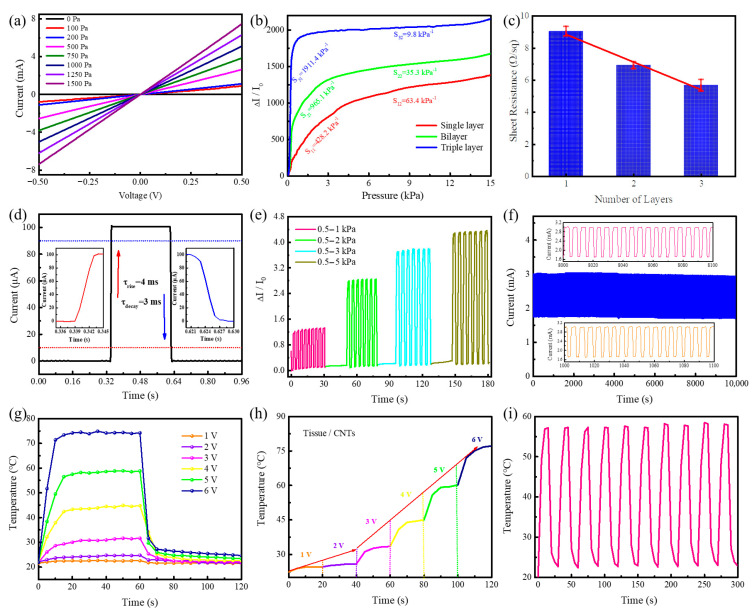
Pressure sensing and electrothermal converting of all-tissue-based electronics. (**a**) Current-voltage (I−V) curves of the sensor with various applied pressures. (**b**) Relative current changes and sensitivity of the sensor. (**c**) Sheet resistance of various tissue layers. (**d**) Response times (rise time and decay time) of the sensor. (**e**) Current-time (I–t) curves of the sensor under serial continuous pressure changes. (**f**) The durability test of the sensor in a pressure range of 1–2 kPa. (**g**) Time-dependent temperature variation with applied voltage. (**h**) Temperature profiles under a stepwise voltage from 1–6 V. (**i**) Temperature–time curve under a 5 V input voltage for 10 cycles.

The first consideration is whether the pressure sensor complies with Ohm’s law for skin-integrated piezoresistive devices. Figure 3a presents the current-voltage (I−V) curves with voltage scanning from −0.5 to 0.5 V for an all-facial-tissue-based sensor, and the linear dependence of voltage on current at various applied pressures clearly shows that there is good ohmic contact between the top sensitive layer and the bottom printed electrode. The pressure-sensing characteristics of different tissue laminations are shown in Figure 3b. The calculation of sensitivity was derived from the formula S = δ(ΔI/I_0_)/δP, where ΔI refers to the relative change in current, I_0_ refers to the initial current, and δP refers to the change of applied pressure. Apparently, the sensitivity of the sensor can be categorized into two regions: an ultrahigh sensitive region and a normal sensitive region, which are highly dependent on the number of tissue layers. Among them, the triple-layered tissue exhibits an ultrahigh sensitivity of 1911.4 kPa^−1^ (even the single-layered tissue reaches up to 428.4 kPa^−1^), which is the highest value of paper-based pressure sensors. The reasons for the significant differences in the number of layers of sensitive materials are further explored. On the one hand, the microscopic wrinkles on the surface of the tissue lead to the spontaneous formation of microstructure air gaps between layers, and these microgaps intuitively indicate that the sensor is sensitive to small external pressures [41]. The compressive stress was measured to assess the mechanical properties of the composite film (Figure S3). Both of the tissues with and without CNTs were capable of deforming mechanically up to 50% under a pressure of 9 kPa, which indicates that the tissue-based sensor is soft enough and prone to deformation. We define an effective elastic modulus (E_eff_) as the slope of the stress versus the strain plot, which is directly influenced by the morphology, roughness, and material properties of the sensitive layer. Both the intrinsic tissue and the composite showed an approximate elastic modulus (≈20.0 kPa), which was also much lower than that of other flexible materials, such as cotton (≈73.6 kPa) [20] and PDMS (≈200 kPa) [18]. On the other hand, a favorable conductive pathway was formed when the CNTs diffused through the microporous structure and adhered to the fibers of the tissue during the immersion process (Figure 2b,c), and the increase of stacked layers also reduced the square resistance (Figure 3c), thus enhancing the current to realize ultrahigh sensitivity. 

In addition, another important criterion for real-time human–machine interaction through pressure sensors is dynamic response, especially the rise and decay time during the process of applying and withdrawing mechanical pressure. As shown in Figure 3d, the rise and decay time are 4 and 3 ms, respectively. Such a rapid response time is sufficient to respond to continuous external pressures. Hence, various degrees of pressure were applied to the sensor, and the results are presented in Figure 3e, indicating that the tissue-based sensor has good reliability and can intelligently perceive and distinguish pressure changes. Periodic cyclic compression experimental tests were conducted to further explore the remarkable performance robustness of the soft-tissue-based pressure sensor. As shown in Figure 3f, the device demonstrated excellent cyclic stability without any evident fatigue in 10,000 s (≈2000 cycles). Notably, all-tissue-based flexible pressure sensors have the promising potential to resolve the issue of electronic waste depending on their biodegradable ability and outstanding biocompatibility, which is described in the combustion process shown in Figure S4 and Video S1.

The high performance of an all-tissue-based pressure sensor is mainly attributed to the internal porous microstructures of the tissue, the microfolds on the laminated surfaces, the high intrinsic conductivity of CNTs, and the spontaneous formation of stable conduction pathways. The work was compared meticulously with other paper-based pressure sensors as shown in Table 1. Obviously, both sensitivity and response time are the best results currently available based on paper-based pressure sensors, and sensitivity greatly increased about 37 times. 

With the excellent electrical conductivity of CNTs, all-tissue-based pressure sensors demonstrated tremendous intelligent thermotherapy application by utilizing the Joule heating effect. As illustrated in Figure 3g, the tissue and CNT composite films exhibit an obviously electrothermal conversion phenomenon, which can reach the equilibrium temperature and recover the initial state in a very short time (both are less than 10 s). The equilibrium temperature generated in this process is derived from the Joule heating effect, which relies on the applied voltage. The temperature can also be correspondingly varied by continuous regulation of voltage (Figure 3h), indicating that films have excellent adjustability in thermotherapy applications. The cyclic thermal response results at an input voltage of 5 V are shown in Figure 3i. A temperature from 23 to 58 °C can be regulated in 10 cycles, proving the outstanding durability under an ambient condition for long-term use. To further evaluate the relationship between the pressure, temperature, and resistance of the all-tissue-based sensor, the temperature–current curve of the sensor under constant pressure loading was measured (Figure S5a), and the current variation of temperature induced was negligible with respect to the regulation of pressure. The pressure-regulated heating properties of the sensor were characterized as pressure–temperature functions under various voltages (Figure S5b), which indicates that the temperature can be manipulated via mechanical pressure from room temperature (19 °C) to designated application temperatures. Consequently, such sensors, combined with electrothermal conversion, provide a very dominant choice for the popularization and application of skin-integrated electronic products. 

### 3.3. Human Physiological Signals Monitoring and Intelligent Thermally Management

The skin surface of a human is a tactile perception system with unique physiological signal signatures for different parts. Based on their ultrahigh pressure sensitivity, fast response time, and conformal ability, all-facial-tissue-based pressure sensors are capable of monitoring all forms of human physical action and some physiological signals in a fast, real-time, noninvasive, and user-interactive way for applications, such as e-skins, medical treatment, and artificial intelligence. 

**Figure 4:**
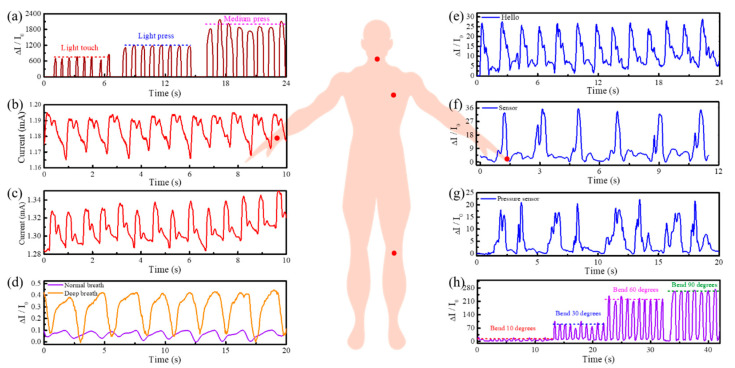
Schematic illustration of human motion and physiological signal monitoring of the pressure sensor. (**a**) Diagram of a sensor touched with various forces. (**b**) Real-time monitoring of the radial artery normal pulse by attaching a sensor to the wrist. (**c**) Exercise pulse test after 1 min of plank. (**d**) Normal breath and deep breath mode test by attaching a sensor to the chest. (**e**–**g**) Voice recognition by mounting a sensor on the throat to detect vocal cord vibrations. (**h**) Detection of leg swing angles by attaching a sensor to the knee.

Generally, it is imperative to maintain the close contact between the pressure sensor and the human skin for subtle monitoring, and the pre-force that exists has almost no effect on the signal acquisition, so the sensor is attached to the corresponding body parts with the aid of a biocompatible woundplast. As presented in Figure 4a–h, the sensor can be installed in various parts of the human body, such as the finger, joints, chest, and throat, indicating a perfect interaction with the human skin. By touching the sensor with different finger forces (Figure 4a), the results showed significant relative changes, whether gently touched or pressed, which facilitates the integration into human skin to distinguish external force information in real time. Joint motion is also detectable, such as knee bending angle. Knee bending tests of 10, 30, 60, and 90 degrees were conducted (Figure 4h), and high current stability was realized by bending the sensor at different angles so that it is highly robust to monitor frequent human motion.

The heart rate (pulse) is often used as one of the important vital signs to assess a person’s physical and mental condition. A large amount of blood entering the arteries increases the pressure and enlarges the diameter of the arteries, which can be felt on the shallow surface of the body, especially the wrist. The pulse beat under normal condition and after 1 min of plank was measured, and the results are shown in Figure 4b,c. The pulse beats changed from 12 in the normal state to 14 in the exercise state, translating into 72 and 84 beats per minute, respectively. Additionally, the current variation increased from 20 to 40 μA with each pulse beating. Hence, the above results show that exercise can enhance the heart rate and intensity, and the sensor has a certain medical diagnostic ability. Furthermore, the frequency waveforms of normal and deep breathing were tested (Figure 4d), and the contraction and relaxation of the chest can be directly captured, which is one of the major applications of e-skins.

Moreover, voice recognition was realized by attaching the sensor to the throat to distinguish various words or phrases, such as “hello,” “sensor,” and “pressure sensor” (Figure 4e–g). Multiple readings of each word propagate a similar characteristic current signal, and the extended phrase of the word can also maintain general consistency. For example, when the phrase “pressure sensor” is said, the word “sensor” has the original two peak current signals, and perhaps the tone level and word conversion process can cause slight signal disturbances. Generally speaking, tissue-based pressure sensors with the ability of voice recognition not only are useful for the voice recovery of people with vocal cord damage but also promote the development human–machine interactions and artificial intelligence. 

**Figure 5:**
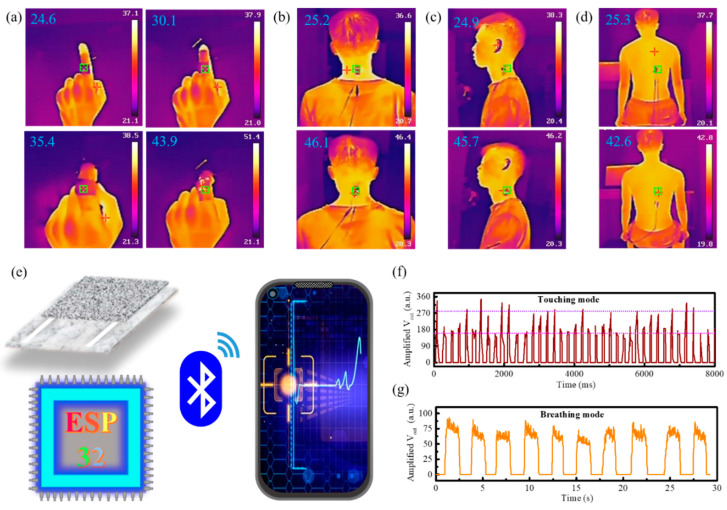
Thermotherapy applications and wireless real-time monitoring of the sensor. (**a**) Temperature dependent with various bending angles. (**b**–**d**) Temperature changes during cervical and lumbar spine twisting. (**e**) Wearable sensor system for wireless transmission and data storage function configuration. (**f**) Wireless function spread on touching mode. (**g**) Respiratory signals of mobile terminal by attaching the device to the chest.

More importantly, adopting a pressure-regulated strategy realizes accurate electrothermal conversion, enhancing the great possibility of providing wearable thermotherapy (the therapeutic effect induced by heat), that is, rehabilitation of rheumatoid arthritis, regulation of joint stiffness, and improvement of blood circulation. Figure 5a depicts the self-regulating form of temperature at various knuckle bending angles. Variable bending angles promote electrical contact enhancement to generate more Joule heat. This effect intuitively translates into a change in temperature. The heat generation of wearable electronics through the continuous movement of joints can achieve the effects of relaxing muscles and activating blood circulation in two ways (joint motion, thermotherapy), which is conducive to the rehabilitation of rheumatoid arthritis. Such wearable pressure sensors can also be used on important body parts, such as the cervical vertebra and lumbar vertebra (Figure 5b–d). For example, twisting the neck or moving the waist can realize the function of automatic thermotherapy. Consequently, wearable electronics can greatly relieve our work fatigue and prevent cervical spondylosis and other problems. 

Through the assembly of a tissue-based sensor and multifunctional integrated module ESP32 (Bluetooth, antenna, filter, power source, etc.) and programmed software writing and debugging, the wireless transmission and signal acquisition of portable smartphones were successfully realized (Figure S6). The auto-stored data of pressure-applied and respiration process from the previous moment were taken out and are depicted in Figure 5e–g. On the one hand, the random variation of pressure signals on touch mode indicates the controllability of the sensing system, and this sensor can wirelessly feedback the pressure condition of the external environment. On the other hand, 10 breaths in 30 s indicates some basic physical conditions of the human body, and such sensor can also wirelessly monitor physiological signals in real time. The continuous breath process is shown in Video S2. Consequently, the systematic implementation of wireless data transmission and storage provides a crucial step for skin-integrated electronics, and the real-time feedback function lays a solid foundation for soft robotics, medical diagnostics, and artificial intelligence applications.

## 4. Conclusions

In this paper, pressure sensors with ultrahigh sensitivity were prepared by using an all-tissue-based backbone and integrating them with a Bluetooth module for wireless transmission. In particular, these sensors offer a sensitivity of up to 1911.4 kPa^−1^, fast response time, outstanding reliability, and environmental degradability. Such all-tissue-based pressure sensors can be of value in many applications, including basic human motion detection, pulse detection, respiratory detection, and voice recognition. Through the thermal effect of pressure regulation, wearable thermotherapy electronics for protecting against rheumatoid arthritis and cervical spondylosis were further realized. It is anticipated that the sensor can easily provide a more scalable method for skin-integrated electronics to manufacture large-area thermotherapy pressure sensors. 

## Figures and Tables

**Table 1:** Comparison of all research studies about paper-based pressure sensors.

Device Types	Active Materials	Method	Sensitivity	Response Time	Ref
Resistive	PEDOT:PSS	Dip coating	1.14 kPa^−1^	20 ms	[38]
Resistive	Silver nanowires	Dip drying	1.5 kPa^−1^	/	[39]
Resistive	Single-wall CNT	Stacking	2.2 kPa^−1^	35 ms	[40]
Resistive	MXene nanosheets	Soaking	3.81 kPa^−1^	11 ms	[42]
Resistive	Gold nanowires	Dip coating	7.38 kPa^−1^	17 ms	[27]
Resistive	Graphene	Soaking	17.2 kPa^−1^	120 ms	[41]
Resistive	Carbon black	Drop casting	51.23 kPa^−1^	200 ms	[43]
Resistive	Conductive rubber	Screen printing	/	600 ms	[44]
Capacitive	Pencil trace	Drawing	0.63 kPa^−1^	180 ms	[45]
Capacitive	Silver nanowire	Airbrush spraying	1.05 kPa^−1^	/	[46]
Resistive	Aqueous solution of CNTs	Immersing	1911.4 kPa^−1^	4 ms	This work

## Supplementary Materials

The following are available online at https://www.mdpi.com/2079-4991/10/12/2536/s1. Figure S1: (a) Surface topography of tissue in microscope. (b) Bottom electrode after screen printing on tissue. (c,d) Adhesion of silver paste used in bottom electrode; Figure S2: SEM images about the surface morphologies of tissue. (a) Surface distribution. (b) Local distribution. (c) Single fiber distribution; Figure S3: Consecutive compression tests on the sensitive layer. (a) Intrinsic tissue. (b) Tissue and CNT composites; Figure S4: Degradation process of all tissue-based sensors. (a) Before combustion. (b,c) Combustion process. (d) Combustion degradation completed; Figure S5: (a) The temperature-current curve of pressure sensor under constant pressure loading (pressure: 2 kPa, voltage: 3 V). (b) The pressure-temperature functions under varies voltage; Figure S6: Schematic diagram of Bluetooth wireless system; Video S1: Combustion process of all tissue-based sensor; Video S2: Continuous breath process of wireless sensor.
