# Supplementary material for: An Ultrahigh Sensitive Paper-Based Pressure Sensor with Intelligent Thermotherapy for Skin-Integrated Electronics
Source: Nanomaterials (Basel). 2020 Dec 17;10(12):2536. doi: 10.3390/nano10122536 (PMC7765889; doi:10.3390/nano10122536)
Supplement: Supplementary file 1 [file nanomaterials-10-02536-s001.zip › nanomaterials-1040907-SI/SI/Supplementary Materials.docx]

Supplementary Materials

An Ultrahigh Sensitive Paper-Based Pressure Sensor with Intelligent Thermotherapy for Skin-Integrated Electronics

Lin Gao ^1,2^, Junsheng Yu ^1^, Ying Li ^2,^*, Peiwen Wang ^2^, Jun Shu ^2^, Xiaoyan Deng ^2^ and Lu Li ^1,2,^*

^1^ State Key Laboratory of Electronic Thin Films and Integrated Devices, School of Optoelectronic Science and Engineering, University of Electronic Science and Technology of China (UESTC), Jianshe North Road, Chengdu 610054, China; lin_gaoedu@163.com (L.G.); jsyu@uestc.edu.cn (J.Y.)

^2^ Research Institute for New Materials Technology, Chongqing University of Arts and Sciences,
Honghe Avenue, Chongqing 402160, China; pwenwang@163.com (P.W.); s917549966@163.com (J.S.); dxy151234@163.com (X.D.)

***** Correspondence: leoyingchem@cqwu.edu.cn (Y.L.); lli@cqwu.edu.cn (L.L.)


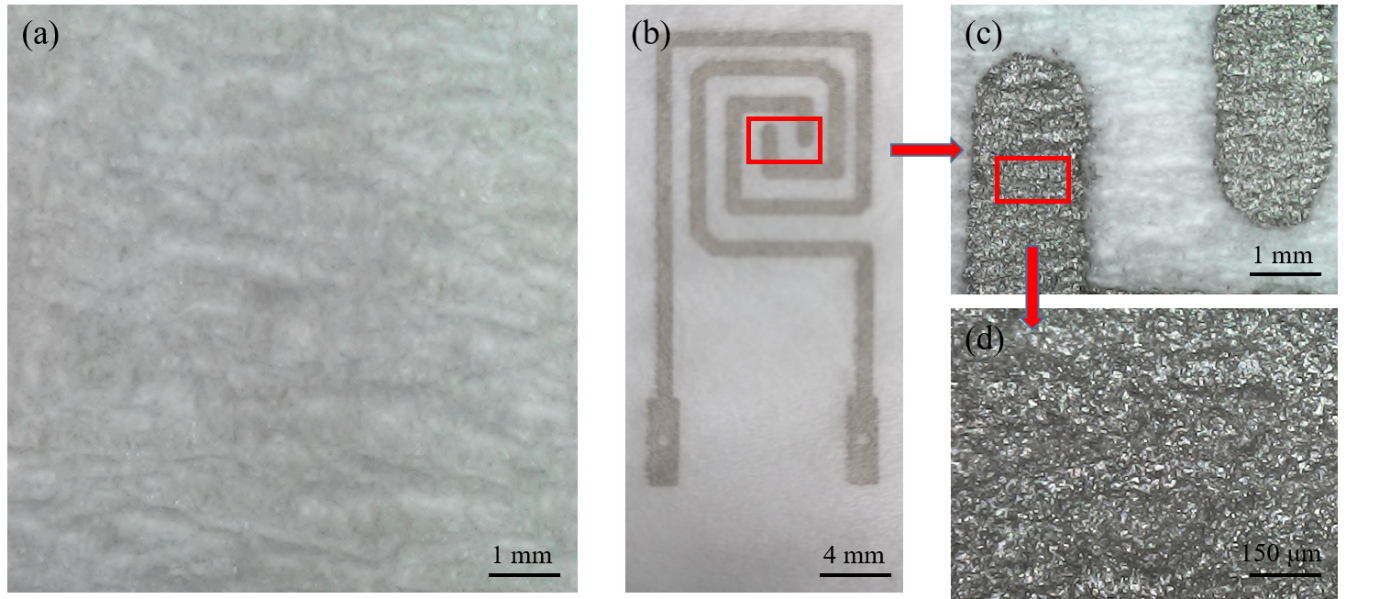


**Figure S1.** (**a**) Surface topography of tissue in microscope. (**b**) Bottom electrode after screen printing on tissue. (**c,d**) Adhesion of silver paste used in bottom electrode.


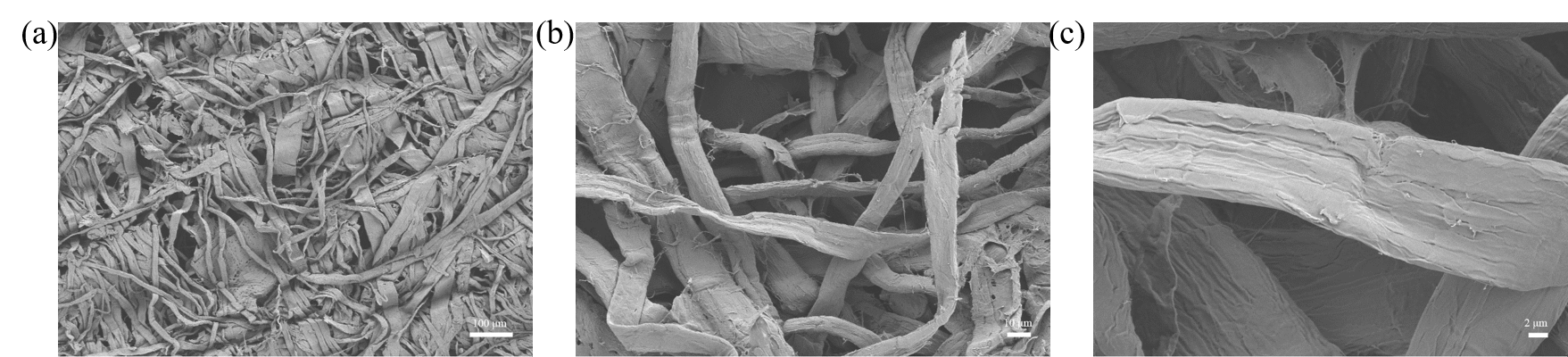


**Figure S2.** SEM images about the surface morphologies of tissue. (**a**) Surface distribution. (**b**) Local distribution. (**c**) Single fiber distribution.


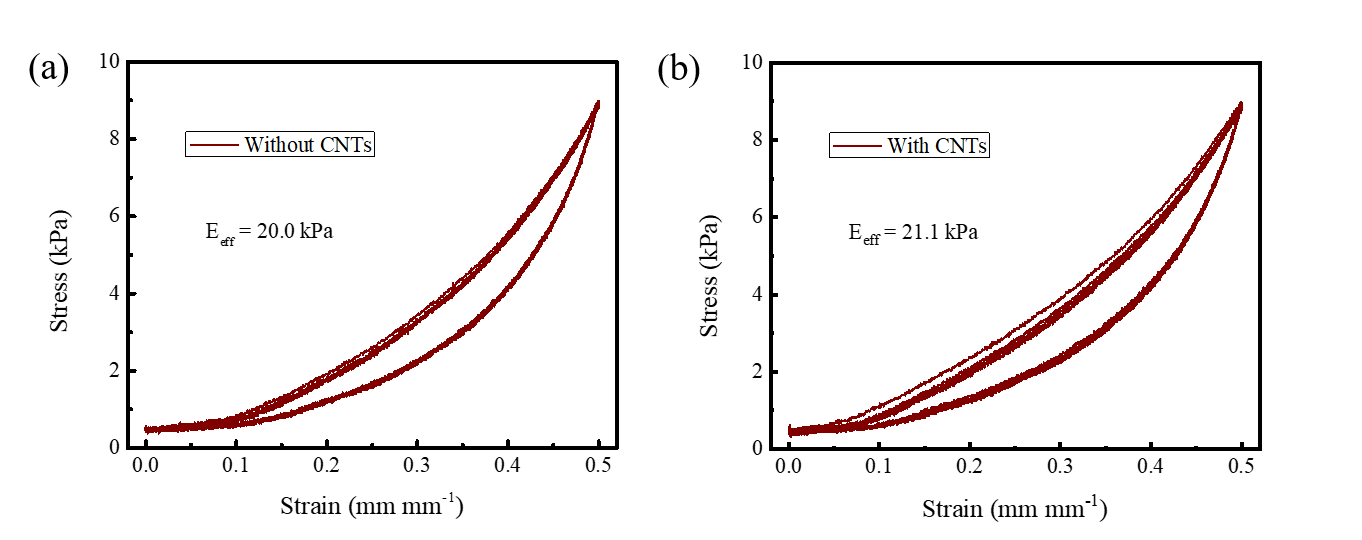


**Figure S3.** Consecutive compression tests on the sensitive layer. (**a**) Intrinsic tissue. (**b**) Tissue and CNT composites.


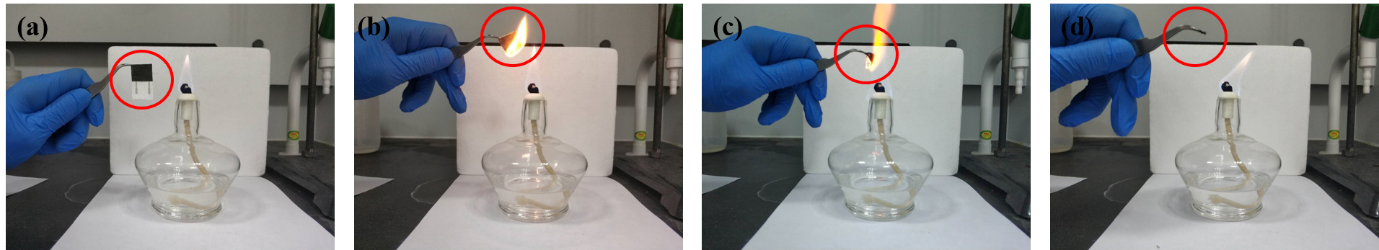


**Figure S4.** Degradation process of all tissue-based sensors. (**a**) Before combustion. (**b**,**c**) Combustion process. (**d**) Combustion degradation completed.


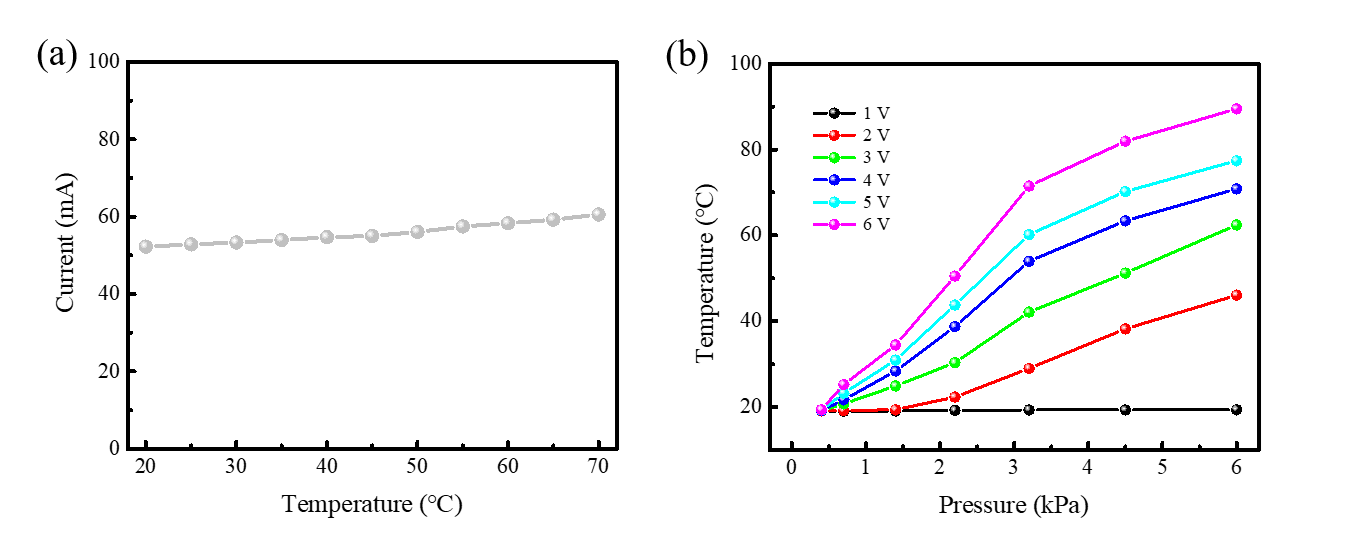


**Figure S5.** (**a**) The temperature-current curve of pressure sensor under constant pressure loading (pressure: 2 kPa, voltage: 3 V). (**b**) The pressure-temperature functions under varies voltage.


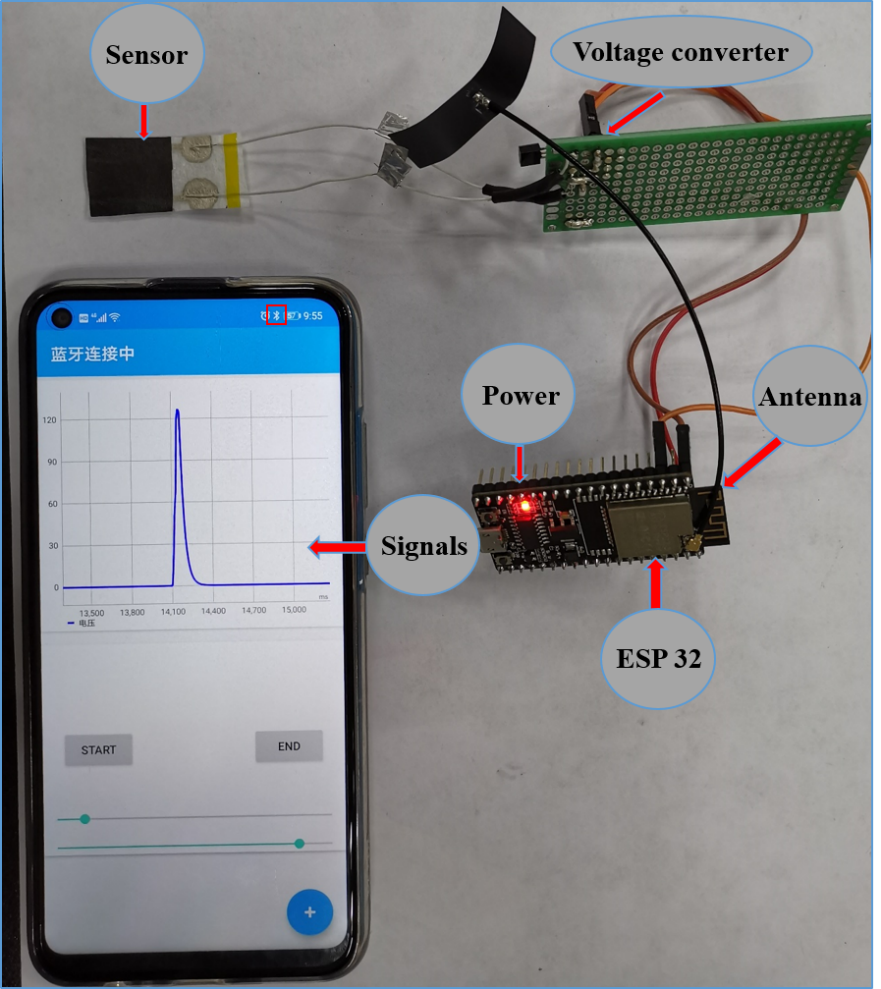


**Figure S6.** Schematic diagram of Bluetooth wireless system.
